# Go Zika Go: A Feasibility Protocol of a Modified Ride-on Car Intervention for Children with Congenital Zika Syndrome in Brazil

**DOI:** 10.3390/ijerph17186875

**Published:** 2020-09-21

**Authors:** Egmar Longo, Ana Carolina De Campos, Amanda Spinola Barreto, Dinara Laiana de Lima Nascimento Coutinho, Monique Leite Galvão Coelho, Carolina Corsi, Karolinne Souza Monteiro, Samuel Wood Logan

**Affiliations:** 1 Postgraduate Program in Rehabilitation Sciences and Postgraduate Program in Collective Health, Federal University of Rio Grande do Norte—Faculty of Health Sciences of Trairi (UFRN-FACISA), Santa Cruz 59200-000, Brazil; smkarolinne@gmail.com; 2 Department of Physical Therapy, Federal University of São Carlos, São Carlos 13565-905, Brazil; accampos@ufscar.br (A.C.D.C.); carol.corsi7@gmail.com (C.C.); 3 Postgraduate Program in Rehabilitation Sciences, Federal University of Rio Grande do Norte—Faculty of Health Sciences of Trairi (UFRN-FACISA), Santa Cruz 59200-000, Brazil; amandaspinola@gmail.com (A.S.B.); dinaralaiana@hotmail.com (D.L.d.L.N.C.); 4 Postgraduate Program in Collective Health, Federal University of Rio Grande do Norte—Faculty of Health Sciences of Trairi (UFRN-FACISA), Santa Cruz 59200-000, Brazil; monique.masso@gmail.com; 5 College of Public Health and Human Sciences, Oregon State University, Corvallis, OR 97331, USA; sam.logan@oregonstate.edu

**Keywords:** congenital Zika syndrome, user participation, mobility, intervention

## Abstract

Children with congenital Zika syndrome (CZS) present severe motor disability and can benefit from early powered mobility. The Go Zika Go project uses modified ride-on toy cars, which may advance the body functions, activities, and participation of children. This paper describes the study protocol aiming to assess the feasibility of a modified ride-on car intervention for children with CZS in Brazil. A mixed-methods design with a multiple 1-week baseline, 3-month intervention, and 1-month follow-up will be implemented. Modified ride-on car training sessions will be conducted three times a week at the participants’ home or in the clinic. The primary outcome will be a narrative description of study feasibility (photovoice method, focus groups, parent feasibility questionnaire and assessment of learning powered mobility). Secondary outcomes will be switch activation, driving sessions journal, social-cognitive interactions, mobility (pediatric evaluation of disability inventory computer adaptive test), goal attainment scaling (GAS), and participation (young children’s participation and environment measure). Go Zika Go is expected to be viable and to improve function, activity, and participation of children with CZS, providing a low-cost, evidence-based rehabilitation option that will be relevant to early child development in a global perspective.

## 1. Introduction

Congenital Zika syndrome (CZS) became a global concern after the Zika virus outbreak in 2015, which mainly affected Brazil [1]. The situation is more serious in the Northeast region, which concentrates the majority of cases, and is marked by strong social problems, scarcity of investments in the training of human resources and in the provision of health services [2]. Children with CZS have severe motor disability, the majority of them compatible with Gross Motor Function Classification System (GMFCS) classification V (i.e., no prognosis for independent mobility) [3]. Only one study evaluated the profile of functioning of children with CZS in Brazil, demonstrating that activity and participation were highly impacted, and that societal attitude was the main barrier to participation [4]. To date, only one preliminary study has been published on the rehabilitation of children with CZS between 3 and 9 months of age. The results indicated that the intervention program based on the principles of Goals-Activity-Motor Enrichment (GAME) improved mothers’ assessment of their babies’ performance, and satisfaction with the performance of functional priorities and the perception of an enriched home environment [5]. 

As CZS is a completely new condition, rehabilitation approaches more commonly used are based on recommendations for cerebral palsy (CP). Even for CP, evidence-based interventions are more easily available for children with mild to moderate motor impairment, and interventions are limited for children with severe motor impairment [2,6]. Young children with typical development exhibit gains in cognitive development, communication, and social skills after starting independent walking [7,8,9]. Young children with motor disabilities are often not able to take part in self-initiated mobility, and are more likely to experience cognitive and developmental delays, as well as less social interactions with caregivers and peers [10,11].

For young children with disabilities, early powered mobility may advance the body function, activity, and participation, in contrast to other interventions within pediatric rehabilitation which are often focused exclusively on one physical skill (body structure domain) in isolation of other therapeutic goals within a stagnant clinical environment (ex. treadmill training). Research indicates positive results of powered mobility interventions for young children with functional impairments [11,12] since battery-operated ride-on cars are easily modified and are considered as an option of increasing interest for motorized mobility for children with disabilities.

Globally, there are very few commercially available motorized wheelchairs for young children with disabilities and existing options are extremely expensive (>USD 17,000 for a base model) [13]. Environmental inaccessibility and device characteristics inhibits motorized wheelchair use [12,13,14,15].

The low cost of modified cars (<USD 400) can minimize some of the barriers previously reported, such as an inaccessible physical environment, financial impact, and peculiarities of motorized wheelchairs readily available commercially. The modifications are fundamental and include the use of large and easy-to-press actuators generally positioned on the steering wheel; in addition to the requirements to provide stability in the seat using common materials, such as polyvinyl chloride (PVC) pipe, swimming kickboards, and Velcro. 

Several studies have showed the results of a powered mobility intervention with modified ride-on car on the behavior and development of young children with disabilities, including CP. Although none of the children in these studies were formally referred to use powered mobility devices due to their young age or diagnosis, all demonstrated the ability to independently press the activation switch, enjoyed driving sessions, and some experienced increased self-care mobility [16], social skills [16,17], as well as increased peer interaction on the playground [18], and during an inclusive playgroup [19]. The limitations of previous work includes a heavy reliance on low-evidence level research designs (i.e., case reports and case series); the inclusion of participants with varying disabilities in the same sample; and the reporting of a wide variety and often very low adherence rates to recommended use guidelines. These limitations have hindered the interpretation and generalization of study results and will be addressed in the current protocol. 

Although participation-based interventions are a promising strategy not only to improve participation but also body functions [20], there are no evidenced-based practices to guide clinical recommendations of early powered mobility device use, including modified ride-on cars, for young children with disabilities, including CZS. The current protocol will address this gap in knowledge and will have a direct and immediate impact on clinical practice. From a global health standpoint, the results may provide an evidence-based rehabilitation option that will be relevant to early child development in low resource contexts. The objective of this study is to determine the feasibility of a powered mobility intervention for young children with CZS with severe motor impairment including acceptability and effectiveness. The specific aims are:

- To evaluate the acceptability of a modified ride-on car intervention for young children with CZS and their families.
- To determine the effect of a modified ride-on car intervention on the (a) mobility and social function, and (b) participation of young children with CZS and their families.
- To understand the barriers and facilitators of a modified ride-on car intervention for young children with CZS and their families.

## 2. Materials and Methods

### 2.1. Design

**Figure 1:**
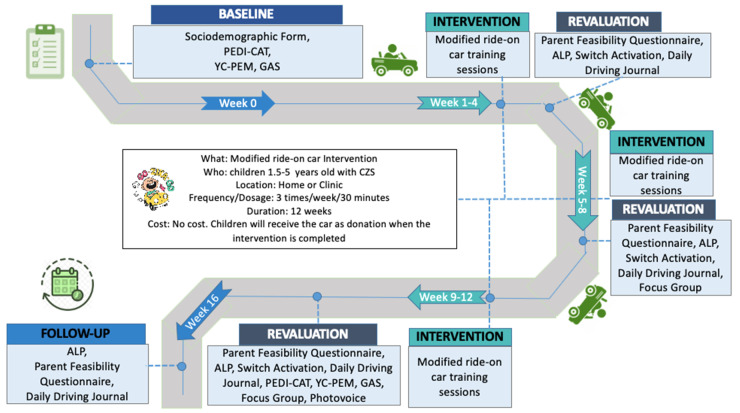
Go Zika Go timeline and procedure intervention for children with congenital Zika syndrome (CZS) between 1.5–5 years age.

This is a feasibility trial of a mixed-methods case series, prospectively registered, with 3-month intervention and 1-month follow-up. The follow-up will aim to assess preliminary learning retention and implications of modified ride-on car training sessions [16]. This prospective study will provide important information about the feasibility, assistive effectiveness, and rehabilitative effectiveness of the first intervention focusing on powered mobility for children with CZS in Brazil. The procedures and timeline of the study are included in Figure 1 and Table 1.

### 2.2. Participants

Inclusion criteria: (a) Diagnosis of CZS, (b) GMFCS [21], level IV or V, (c) aged between 1.5 and 5 years, (d) no previous experience with powered mobility. 

Exclusion criteria include the presence of uncontrolled seizures and musculoskeletal deformities that prevent adaptation of the seating support in the device. 

### 2.3. Sample Size Estimation

In this feasibility study, we will recruit 10 children with CZS. This small sample will provide data on the feasibility of the protocol, thus helping determine the sample size for a larger randomized trial using powered mobility in children with CZS. Given the lack of previous studies measuring the outcomes of interest for this study, the sample size was estimated according to recommendations of the minimum sample for pilot studies [22].

### 2.4. Settings and Locations Where the Data Will Be Collected

The scenario of CZS in Brazil is 3523 confirmed cases, with the Northeast region being the most affected, with 1907 cases. Rio Grande do Norte, where this study will occur, is one of the 9 states in the Northeast region and has 126 confirmed cases of CZS. In the national gross income ranks, it occupies the 17th position among the 34 Brazilian states. In 2010, the Human Development Index of Rio Grande do Norte was 0.684 and ranked 16th among the 27 federative units in the country. 

Recruitment of participants will take place through early intervention services, pediatric clinics that provide physical and occupational services, and community-based programs that serve the study populations throughout Rio Grande do Norte (RN) state in Brazil. The recruitment will take place between January and December 2021.

### 2.5. Patient and Public Involvement

Parents’ involvement in the design of the protocol: parents or caregivers of children diagnosed with CZS who received a rehabilitation service at Clínica Escola de Fisioterapia da Facisa—Santa Cruz, RN were asked to answer a questionnaire prepared by the researchers, with 8 questions related to intervention preferences. Initially, the parents watched a video about the Go Zika Go project and then they were interviewed by the first author (EL) at the clinic or at home. The questions included aspects of the families’ interest in participating in the intervention, space available at home to use the ride-on car, ideal time for mobility training, preference for the place to perform the intervention, etc. Five parents/caregivers participated in this exploratory stage of the research. The results of this stage showed that all families were interested in participating in the intervention and stated that the training can be performed at home and/or at the clinic setting.

### 2.6. Outcomes Measures

#### 2.6.1. Primary Outcome

The primary outcome of the study will be the feasibility of the protocol, including a narrative description, recruitment and retention rates, adherence and acceptability of the protocol. A mixed methods approach will be used to assess the primary outcome.

A document analysis will determine the number of parents who received information about the study by mail, the number of potential participants contacted by telephone, how many parents met the inclusion criteria and how many provided informed consent, received, and completed the intervention. 

Qualitative data will be collected using the photovoice method and focus groups. Photovoice is a participatory action-research modality that uses photographs to express individuals’ experiences during their daily realities [23].

Photovoice aims to record and present everyday realities using photography; promote dialogue and critical reflection of reality and know the strengths and weaknesses of the target audience; and reach decision makers [24]. Photovoice will explore families’ perception of the feasibility of modified ride-on cars for children with CZS at home and in the community. In the eighth week of the intervention, the parents of children with CZS will be invited to participate in a focus group to provide information about the application of photovoice, its ethical aspects, to clarify doubts about the handling of cameras, expectations in participation and to solve personal issues that may arise. Thus, the methods will follow the Wang and Burris [25] recommendations. Disposable cameras will be offered to record the photos. Parents or caregivers who prefer not to use the camera offered by the researchers will use the cameras on their smartphones and share information by the messaging app. Guidance will be provided on how to capture quality photos, and information about the privacy of people in the scene to be photographed. Parents/caregivers will be instructed to photograph moments of the child’s routine at home and in the community. To guide the capture of the photos, parents/caregivers should follow a script of questions asked by the researchers, which include: (1) How does my child feel when using the modified ride-on car? (2) Does the modified ride-on car help my child to get around at home and in the community? (3) Does the modified ride-on car help my child to play with other children? When and where is the modified ride-on car used? Participants will be instructed to capture photos that answer study questions over the next 4 weeks (the third month of intervention). It is recommended that each participant register between 20 and 30 photos [25]. After this period, digital images or printed photographs will be delivered to the research team for the preparation of the second focus group. 

In the 12th week of the intervention, the second focus group will be held, where the photos will be displayed and shared with the researchers and other participants, so that the images can be discussed, allowing for a deep reflection from the creation of a proper reading of each moment, on the use of modified ride-on cars by children with CZS at home and in the community. Each participant will be asked to select the most significant pictures representing the experience of their children using the ride-on car. The showed method [26] will be used through its specific questions to facilitate assistance to participants in contextualizing the meaning of their photographs. In addition to the discussion on the contents of the photos, the following feasibility questions guide will be used: What is your perception of the intervention with modified ride-on cars? What helped or hindered your child’s use of the modified ride-on cars at home and in the community? Do you think the intervention was useful for your child? All focus groups will be conducted in person by two members of the research team. The sessions will be recorded in audio and video, transcribed and analyzed thematically [27,28]. 

Quantitative data will be collected from feasibility questionnaires for parents and professionals. The parents feasibility questionnaire (PFQ) [29,30] includes questions related to satisfaction with the intervention, the time of use at home and in the community, the usefulness of the ride-on car, the level of comfort related to the time of its use and the need for help from others during use. PFQ will be used at the end of week 4, 8, and 12 of the intervention, and at follow-up. The assessment of learning powered mobility (ALP) [31] will be used to measure the feasibility of the intervention. This tool is able to describe the learning process’ phase at the initial stages of using a powered mobility device. It also documents the child’s occupational performance, thus supporting the selection of learning strategies. Phases are scored from 1 (novice) to 8 (expert) to describe occupational performance while using a powered mobility device, considering five categories: understanding of tool use, level of attention, expressions and emotions, activity and movement, and interaction and communication [31,32]. In this study, the ALP will be applied at the end of weeks 4, 8, and 12 of the intervention, and at the follow-up. In addition, a sociodemographic questionnaire will be used for all participants at the baseline.

#### 2.6.2. Secondary Outcomes

##### Switch Activation

Switch activation is defined as when the switch is pressed to activate the modified ride-on car and make it “go”, thereby moving in space from one place to another [30]. Switch activation may occur from the child in the modified ride-on car independently or with assistance (either physical or verbal cueing) from others. Assistance may be provided by a sibling or peer, or an adult such as a parent/caregiver or clinician. The duration of switch activation, who activated the switch, and the level of assistance, if any, provided to the child using the modified ride-on car will be coded. Total switch activation time (minutes and seconds) and percentage of time of each 10-min video will be coded and reported. Switch activation analyses will be carried out at the end of week 4, 8, and 12 of the intervention.

Inter- and intra-rater reliability (at least 85% agreement) will be established on 10% of the video recordings amongst one expert coder and two secondary coders. Training will include coding of video recordings, discussion of disagreements, and coding of additional videos until reliability is established. All videos will be coded in Datavyu.

##### Daily Driving Journal

Parents will record the date, duration (minutes) location, and activities during each driving session in a daily driving journal. The researcher will collect the daily driving journal at the end of the study. The daily driving journal for entries of week 4, 8, and 12 of the intervention, and in the follow-up will be analyzed.

##### Pediatric Evaluation of Disability Inventory–Computer Adaptive-test (PEDI-CAT)

The PEDI-CAT is the computerized version of PEDI and has been translated and adapted culturally to Brazil [33]. It is answered by the children’s caregiver and has an item bank divided into two domains: (1) mobility, which includes 75 items ranging from basic motor skills (e.g., sitting without support) to more difficult motor skills (e.g., running or climbing a step ladder). Additionally, this domain includes the use of walking devices; (2) cognitive/social, which includes 60 items related to interaction (e.g., follows the gaze of another person), communication (e.g., uses gestures to ask for something), everyday cognition (e.g., recognizes his/her name), and self-management (e.g., when upset response without hitting). In these domains, the four-point scores (unable, hard, a little hard, easy) are based on different levels of difficulty. The overall score is transformed in a normative score (based on age) and a continuous score that will be used in the analyses. The PEDI-CAT will be administered on the baseline and at the end of the 12th week of the intervention.

##### Young Children’s Participation and Environment Measure (YC-PEM)

The YC-PEM, aimed to be used for children from zero to five years, explores the frequency of participation in activities, the level of involvement of participation, the parent’s satisfaction with the children’s current participation and the environmental supports and barriers considered to be important. Parents will report their child’s participation across home, community and childcare activities. For each type of activity, parents will report on (1) frequency of attendance (i.e., how often the child attends an activity) (8-point scale, from never (0) to once or more times every day (7); (2) level of involvement (i.e., the child’s level of engagement in the activity) (5-point scale, from not very involved (1) to very involved (5)); and (3) their desire for their child’s participation to change (i.e., their dissatisfaction) [yes, no]. Then, caregivers will evaluate the impact of environmental features and resources on participation (3-point scale, from usually helps/usually yes (3) to usually makes harder/usually no (1)). The YC-PEM will be administered at baseline and at the end of the 12th week of the intervention [34].

##### Goal Attainment Scaling (GAS)

GAS is an objective method of quantifying goal attainment. Goals are scored on a Likert-type scale from −2 (representing no positive change at all from baseline/ regression), −1 (a little less change than expected), 0 (attainment of goal at the expected level), +1 (a little more change than expected), to +2 (attainment of goal at much more than the expected level) [35]. Goals are personally relevant to the individual family (rather than standardized), with the distance between each increment representing a relatively equal amount of effort or improvement to achieve. Outcome scores on an individual’s goals will be converted to an aggregate T score (regardless of the domain to which the GAS goal is aligned) which will be the unit of analysis. One to three GAS goals will be set in the baseline and at the end of the 8th week of the intervention. At the end of the 12th week of the intervention, the goals will be reviewed.

### 2.7. Intervention

**Figure 2:**
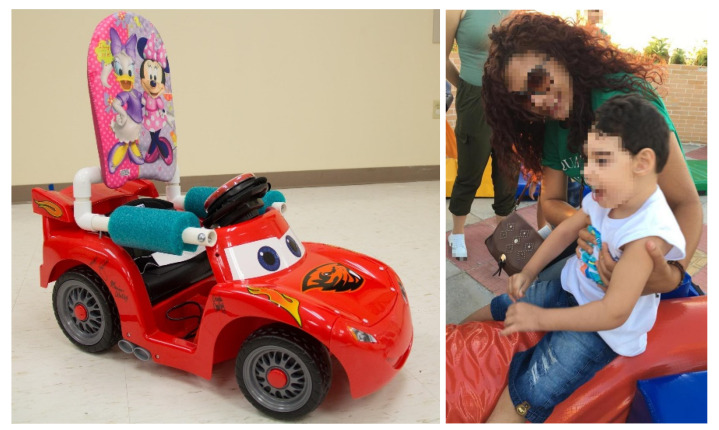
Modified ride-on car and a child with CZS.

Modified ride-on car training sessions will be conducted three times a week at the participants’ home or in the clinic, according to family preference. A therapist will guide the sessions at both locations. The intervention session will include the following: (1) environment setup (e.g., instructing families) (5 min); (2) natural play as a warm up activity (5 min); and (3) social training and mobility with modified ride-on cars (30 min). The focus of treatment sessions will be based on the social function goals and the expected mobility set by caregivers in the modified GAS after the pretest [36]. The 30-minute driving session will involve the participants learning cause–effect concepts by driving the modified ride-on car (i.e., pressing the switch for moving and releasing it for stopping). The dose of the intervention will be adjusted considering the opinion of the parents who participated in a previous interview and the results of a recent study, where the average time of interventions was 24 to 30 min [37]. The therapist and caregivers then will use verbal and physical cueing to encourage children to drive and explore the surrounding environment [17,38]. All sessions will be video- and audio-recorded. Figure 2 shows a modified ride-on car and a child with CZS.

### 2.8. Modified Ride-On Cars

Ride-on cars will be modified with a joystick for activation. Joystick control will allow omni-directional turning and proportional speed-control. The vast majority of research with modified ride-on cars has installed a large, easy-to-press, all-or-nothing activation switch that does not provide turning nor speed control. These steering and speed modifications will enhance the ability for young children with CZS to explore the environment and use the device within the home and community. Joystick activation ensures that children are provided with an opportunity to use an activation method that is consistent with future joystick-driven device options, such as motorized wheelchairs. Ride-on cars will be modified with optimal seating support using readily available, low-cost materials based on each child’s individual needs. Ride-on car models will be selected based on their design and structure to provide seating support. 

### 2.9. Statistical Analysis 

Quantitative data will be imported into the Statistical Package for Social Science (SPSS) 22.0 for analysis and reported using descriptive statistics (absolute and relative frequencies, measures of central tendency and measures of variability). For numerical data, the Wilcoxon test will be used when there are two measurements, while the Friedman test will be used when there are more than two measurements. For categorical data, the Chi-Square test will be used. All the analyses will consider a confidence interval of 95% (CI95%) and a statistical significance of p < 0.05. Adverse events will be categorized. The percentage of scheduled and completed treatment sessions will be compared.

The feasibility results will be presented qualitatively. The researchers will analyze the audio transcripts of the focus group, Photovoice and additional questions through the thematic analysis to identify and validate the significant themes and patterns from the discussion with the parents. The ATLAS.ti software will be used to code, group and present the data, based on the results.

### 2.10. Ethics

This study was approved by the Research Ethics Committee of the Faculty of Health Sciences of Trairi (FACISA), of the Federal University of Rio Grande do Norte (UFRN), under opinion 3.980.703/2020 and CAAE 29582020.8.0000.5568. Written informed consent will be obtained from parent or guardian prior to data collection. The trial was prospectively registered at the Brazilian Registry of Clinical Trials (ReBEC) (RBR-2Y2Z8P). The study follows the Consolidated Standards of Reporting Trials (CONSORT) to pilot or feasibility trial.

### 2.11. Knowledge Translation (KT) and Dissemination

End of grant KT and Integrated KT strategies will be used [39]. An appropriate KT planning template will be used to maximize the scope of the findings, while acknowledging the nature of feasibility studies. Traditional knowledge diffusion strategies will be used as End of grant KT, such as conference presentations and papers publications. A media engagement strategy will disseminate the results in partnership with Nossa Casa, a NOG Brazilian institution responsible for the main actions of KT in Brazil on childhood disability. The results will also be shared at the REDE’s website, which is an international network aiming to build research capacity as well as preparedness regarding emerging infectious diseases in Latin America and Caribbean. A video and an infographic will be made with the results of the study in plain language, which will be distributed in services and public centers that care for children with CZS. Integrated KT will involve stakeholders, families and researchers, assuring that the outcomes meet the needs of the end users. A workshop will be organized with both activities for the dissemination of results and building capacity on modification of ride-on cars. The final results will also be transformed into a policy brief and forwarded to public services and sectors. The dissemination workshop will be hosted in collaboration with families, the local research team members and international collaborators.

## 3. Potential Contributions of This Study

This study will fill an important research gap and may contribute to future intervention studies, having a direct and immediate impact on clinical practice in children with CZS. This study protocol will determine the feasibility of an intervention using powered mobility for young children with CZS, who present severe motor impairments. The acceptability of the intervention for young children with CZS and their families will be described. In addition, we will assess the effects of the intervention on mobility, social function and participation of children; the barriers and facilitators of the intervention will also be analyzed.

Feasibility studies are relevant as they use rigorous methods to access processes, resources, management and effects related of the intervention of interest [40]. The feasibility assessment includes data on the acceptability of the intervention and the perceived burden of the procedures. Feasibility studies may therefore contribute resources invested in larger trials that are the most likely to generate clinically significant results [41]. In this protocol, we propose an approach of mixed methods; the primary outcomes will be qualitative, which will allow a deeper understanding in terms of feasibility. The quantitative measures will enable a complementary analysis of the feasibility and contribute to answer the specific objectives of the study. Thus, data obtained in this study will be able to guide future clinical trials on powered mobility in children with CZS and benefit these children and their families. It is expected that this innovative study will contribute evidence for the adoption of low cost environmental early child interventions in Brazil and other low-and middle-income countries around the world.

## Figures and Tables

**Table 1:** Study design schedule.

Assessment Tools	Study Period						
	Enrolment	Baseline	Intervention	Revaluation			Follow up
**Time Point**	**Week 0**	**Week 0**	**Weeks 1–12**	**Week 4**	**Week 8**	**Week 12**	**Week 16**
Eligibility screening	X						
Informed consent	X						
Modified ride-on car training sessions			X				
Sociodemographic Form		X					
PEDI-CAT		X				X	
YC-PEM		X				X	
GAS		X			X	X	
PFQ				X	X	X	X
ALP				X	X	X	X
Switch Activation				X	X	X	
Daily Driving Journal				X	X	X	X
Focus Group					X	X	
Photovoice						X	

PEDI-CAT: Pediatric Evaluation of Disability Inventory–Computer Adaptive-test; YC-PEM: Young Children’s Participation and Environment Measure; GAS: Goal Attainment Scaling; PFQ: Parents Feasibility Questionnaire; ALP: Assessment of Learning Powered Mobility.
